# Korean Version of the 17-Item Utrecht Work Engagement Scale for University Students: A Validity and Reliability Study

**DOI:** 10.3390/healthcare10040642

**Published:** 2022-03-29

**Authors:** Aeri Jang, Minjeong An

**Affiliations:** 1 Department of Nursing, Nambu University, Nambudae-gil, Gwangsan-gu, Gwangju 62271, Korea; sseillerjjang79@gmail.com; 2 College of Nursing, Chonnam National University, 160 Baekseo-ro, Dong-gu, Gwangju 61469, Korea

**Keywords:** engagement, reliability, validity, students

## Abstract

Work engagement is a factor that has key influence on learning outcomes for college students. The psychometric properties of the Korean version of the 17-item Utrecht work engagement scale for students (UWES-S) survey have yet to be determined. The purpose of this study was to test the reliability and validity of the Korean version of the 17-item UWES-S among Korean college students. A total of 248 college students were recruited from three universities in South Korea. Data were analyzed using IBM SPSS and the Mplus program. Among 248 college students, the mean age was 21.19 years, and 87.5% of the students were female. Reliability was deemed satisfactory by Cronbach’s alpha 0.940, McDonald’s omega 0.941, and composite reliability 0.941. Construct validity was supported by confirmatory factor analysis results (CFI = 0.93, TLI = 0.91, SRMR = 0.05, RMSEA = 0.08). Convergent validity was supported by the significant relationship between academic engagement and burnout (r = −0.344, *p* < 0.001). Our findings showed that the Korean version of the 17-item UWES-S was a reliable and valid instrument. This instrument can be used to assess and improve work engagement in college students.

## 1. Introduction

“Work engagement” refers to organization members applying themselves to their work roles. In engagement, people employ and express themselves physically, cognitively, emotionally, and mentally during role performances [1]. “Work engagement” is different from “flow”, which means an experience that lasts for a short period of time [2], and is a concept independent of “burnout” [3,4]. The increased interest in these terms has occurred with the advent of positive psychology [5]. Due to their lower work engagement levels compared with the overall population, students should be regarded as an important group to target [6]. Therefore, for students, “work engagement” should be considered as a meaningful variable; however, it is constituted of an internal mindset that is difficult to recognize or assess.

Schaufeli and Bakker developed a manual of the 17-item Work Engagement Scale (17-item UWES) comprising three domains: vigor, dedication, and absorption at Utrecht university [7]. Moreover, a 17-item UWES-S was conducted in Spain, Portugal, and the Netherlands [8]. In Sri Lanka, India, Japan, and China [9,10,11,12], studies have been conducted to validate the 17-item UWES-S instrument. For Korean students, work engagement has a double-mediated effect on the relationship between mastery goal orientation and burnout [13] and the effect on self-directed learning ability [14], among many others. Research has identified that the UWES-S instrument has key influence on several variables, such as progress. The instrument used in these studies was the 17-item UWES-S [8], and for our research the English version was translated into Korean.

To apply the original instrument to another culture, the linguistic meanings and psychological/cultural differences between the original culture and the target culture must be considered. Moreover, UWES-S serves as a guideline for the development, execution, and evaluation of tests intended for use in groups other than the test group of the original instrument. Adaptation must be conducted in accordance with various conditions and guidelines [15]. Therefore, to measure the objective work engagement level for Korean college students, the translated/adapted Korean version of the 17-item UWES-S instrument should be developed based on various conditions and guidelines. 

Therefore, in this study, the 17-item UWES-S [8] instrument was translated and adapted based on the Korean context, in accordance with the stage of the ITC Guidelines for inspection translation and adaptation [15], and reliability. By testing the validity of the 17-item UWES-S, this study aimed to provide an instrument for creating reliable and valid measures when evaluating future academic engagement for Korean college students. The purpose of this study was to test the validity and reliability of the Korean version of the 17-item UWES-S.

## 2. Materials and Methods

### 2.1. Study Design and Participants

This was a methodological study to translate/adapt the 17-item UWES-S developed by Wilmar Schaufeli and Arnold Bakker into Korean and then verify the validity and reliability of the Korean version of the 17-item UWES-S for Korean college students. 

The study participants were college students enrolled in their respective departments of nursing at various nursing colleges in Korea. Data collection was conducted after creating a list of 15 nursing colleges, selecting three nursing colleges through random selection, visiting three nursing colleges and obtaining consent from the departments of nursing, and explaining the purpose and content of the study. Participants directly filled out the questionnaire. Among the nursing students, those who were on a leave of absence, those aged below 19 (based on the date of participation in the study), and those being treated for a mental illness or acute internal/surgical disease (to control exogenous variables influencing work engagement) within the last six months were excluded. A sample of at least 200 participants is required for the identification of bias probability items in a single-language test [16,17]. Data collected from 248 students were used in the final analysis.

### 2.2. Measures

Work engagement for students was measured using the 17-item UWES-S, which examines student perceptions in various situations related to academic participation [8]. This instrument comprises 17 items in three domains: vigor (VI: 6 items), dedication (DE: 5 items), and absorption (AB: 6 items) on a 7-point scale. In a study by Schaufeli et al. [8], Cronbach’s ⍺ was 0.92, whereas Cronbach’s ⍺ was 0.94 in this study. This indicates that high participation was noted in this study.

To verify the criterion validity of the Korean version of work engagement for students, academic burnout was measured using the Korean academic burnout scale developed and validated by Lee et al. [17]. This instrument comprises five factors: exhaustion, incapacity, antipathy, apathy, and anxiety. On a 5-point Likert scale with a total of 25 questions, a higher score indicates a higher degree of academic burnout. In a study by Lee et al. [17], Cronbach’s ⍺ was 0.92, whereas Cronbach’s ⍺ was 0.91 in this study.

### 2.3. Development of the Korean Version of the 17-Item UWES-S

Before the full-scale translation/translation process began, official approval for the use of the instrument (ITC Guideline 1) was obtained from Schaufeli et al. [8], the developer of the 17-item UWES-S used in this study. To evaluate whether the definition and content of the constructs were sufficiently reflected in the questions (ITC Guideline 2), the English version of the 17-item UWES-S was translated into Korean by two nursing professors who are fluent in both English and Korean, with Korean as their native language. During this translation process, instead of focusing on translating the meaning of each word into Korean, they ensured there were no problems in conveying the original concept of the sentence (ITC Guideline 3), and considered the concepts of “work” and “study” in this instrument. Items 2 and 7 refer to “learning”, and study and work for other items refer to “studying”. Item 4 was translated/adapted as being indicative of “confidence”.

To minimize the impact of language/cultural differences (ITC Guideline 4) unrelated to the intent of the test in the target group, the translation considered the vocabulary commonly used by Korean college students, and the translated instrument was verified by two college students. The “full” in Item 2 was changed to mean “clear”. “I feel like going to class” in Item 8 was changed to indicate “I want to go to school”. The “persevere” in Item 17 was changed to “be patient”. To ensure that the linguistic, psychological, and cultural differences of the target group were considered in the translation of the completed instrument (ITC Guideline 5), three Korean nursing education experts who had Korean as their native language, had an in-depth knowledge of the culture of college students, academic enthusiasm, and experience and knowledge of evaluation principles, verified the translated/adapted instrument. In Item 1, “I feel like I am bursting with energy”, was noted to have a weaker meaning in Korea; “burst” was changed to indicate “feeling energetic”. Moreover, “very resilient” in Item 15 was changed to “very cope,” from “It is difficult to detach myself from my studies”. In Item 16, “not studying is hard for me,” was also revised. To maximize the suitability of the test alternative in the target group, multiple translation design was used, and “I am enthusiastic” in Item 5 was modified to indicate “I have passion”. 

Three Korean nursing education experts were requested to evaluate the relevance of the target group considering the translation of the lation/adaption (CVI > 0.9). A special education professor proficient in both Korean and English performed the reverse translation without prior information about the original instrument, based on the draft of the written questions. This reverse process was used because translating without prior information can prevent bias and lead to meaningful interpretations. Following this process, an American whose native language was English compared the original and reverse-translated instrument to ensure the meanings were similar (ITC Guideline 6). For items not evaluated to be completely identical, the original instrument translators reviewed and revised the translated Korean items once again and went through a reverse translation process, comparing the reverse-translated English instrument with the original instrument.

Finally, to provide evidence that the instructions and items had similar meanings in all target groups (ITC Guideline 7), a survey was conducted with one Korean college student. After the test was conducted, the student verified that they understood the meaning well and responded to the questionnaire. To ensure that the questions, forms, rating scales, scoring categories, test practices, methods, and other procedures were appropriate for the intended target group, the following question was asked: “Is the sentence format, including the physical arrangement between the two languages, the driving force?” In addition, the translation/reverse translation process of the draft questionnaire was completed by the reviewing question: “Is the emphasis on words or phrases identical to the original question?”

Based on the completed draft questionnaire, a preliminary survey was conducted targeting two nursing college students (ITC Guideline 8). The preliminary survey was conducted one-on-one between the researcher and participants. First, participants were asked to respond to the questionnaire in a self-report format, and it was confirmed that the average response time was 15 min. After the preliminary survey and responses were completed, individual interviews were conducted with the survey respondents. Based on the preliminary survey results, two students experienced some difficulty when responding to “when things do not go well” in Item 17 and “I forget everything else around me” in Item 6. Therefore, a nursing expert and an English Literature professor modified the meaning of “even when things are not resolved” to “I forget everything else around me”. The Korean version of the 17-item UWES-S (KUWES-S) was completed after the final revision (Appendix A).

### 2.4. Data Collection

To conduct this study, after obtaining the approval of the Chonnam National University Research Ethics Review Committee (1040198-160907-HR-079-03), data were collected from October to November 2016, with the consent of participants. The participants received detailed explanations about the study purpose and content. Participants were informed that they could withdraw their participation at any point and were reassured that there would be no repercussions for doing so. Moreover, they were also informed that personally identifiable information would not be collected, and that confidentiality and anonymity were guaranteed. Voluntary written consent was obtained from the participants. Participants were then asked to answer the questionnaire in a separate location where the confidentiality of the questionnaire contents was guaranteed.

### 2.5. Statistical Analysis

The general characteristics of participants were calculated as frequency, percentage, and mean and standard deviation using the Statistical Package for the Social Science program version 26.0. Using Mplus version 8.0, confirmatory factor analysis was performed to assess the dimensionality of the KUWES-S. The model fit of the KUWES-S was determined using the Tucker–Lewis Index (TLI), Comparative Fit Index (CFI), Root Mean Square Error of Approximation (RMSEA), and Standardized Root Mean Square Residual (SRMR). The acceptable fit was determined by cut points of TLI > 0.90, CFI > 0.90, RMSEA ≤ 0.08, and SRMR ≤ 0.08 [18]. Furthermore, convergent validity was tested by examining the relationships between academic burnout and each academic engagement subscale of the KUWES-S. The level of significance was set at a *p*-value of <0.05.

Cronbach’s ⍺ was examined to test the internal consistency reliability of the KUWES-S. Moreover, composition reliability (CR) and McDonald’s omega coefficient [19] were computed to further test reliability for multidimensional structures.

## 3. Results

### 3.1. General Characteristics of Study Participants 

The average age of the participants was 21.19 years old, and 217 participants were women (87.5%). Regarding the grade, 61 participants (24.6%) were college freshmen, 64 participants were sophomores (25.8%), 62 participants were juniors (25%), and 61 participants were seniors (24.6%).

### 3.2. Item Descriptive Analysis of the KUWESS-S

The results of analyzing skewness and kurtosis to check the normality of the data used in this study showed that the absolute value of skewness of all measured variables was 0.58 or less and did not exceed 3, and the absolute value of kurtosis was 0.66 or less and did not exceed 10. Univariate normality was satisfied. In the item homogeneity test, corrected item-total correlation coefficients of each subscale ranged from 0.557 to 0.679 for vigor, 0.637–0.732 for dedication, and 0.514–0.684 for absorption (Table 1).

### 3.3. Construct Validity

Using the confirmatory factor analysis, the KUWES-S was tested with the three-factor structure model and the findings showed an acceptable fit (χ^2^ = 357.578 [df = 116], *p* < 0.001; CFI = 0.901, TLI = 0.884, SRMR = 0.048, RMSEA = 0.092) and the intended model fell slightly short of the minimum acceptable value of TLI > 0.90. Model modification was used to improve model fit using modification indices greater than 20 (Vigor: Item 4 with Item 1, Item 8 with Item 4). In the second confirmatory factor analysis with the modified model, all model fit indices showed better model fit (χ^2^ = 294.858 [df = 114], *p* < 0.001; CFI = 0.925, TLI = 0.910, SRMR = 0.045, RMSEA = 0.081; Table 2). In the testing, all the factor loadings of the three factors (vigor, dedication, and absorption) were acceptable (>0.30) (Table 1).

### 3.4. Convergent Validity

To verify the convergent validity, the correlation between academic engagement and academic burnout was tested. The overall academic engagement had a negative correlation with burnout (r = −0.344, *p* < 0.001). As a result of examining the correlation between burnout and three sub-factors of academic enthusiasm, vigor, dedication, and absorption were negatively correlated with academic burnout, respectively (r = −0.350, *p* < 0.001; r = −0.337, *p* < 0.001; r = −0.290, *p* < 0.001, respectively) (Table 3).

### 3.5. Reliability 

The Cronbach’s α coefficient indicating the internal consistency of the Korean version of UWES-S developed in this study was 0.94 (Vigor, 0.846; Dedication, 0.863; Absorption, 0.830). As the KUWES-S had three dimensions, we computed McDonald’s omega and composite reliability coefficients. The McDonald’s omega and composite reliability coefficients were satisfactory with 0.941, respectively (Table 4).

## 4. Discussion

The work engagement of college students is an internal mindset that is difficult to recognize or assess individually, and appropriate instruments are needed to assess the academic process of college students in Korea to provide an appropriate educational approach for addressing the needs of students. In addition, when translating/adapting the developed instrument, it is necessary to consider not only the linguistic meaning, but also the psychological/cultural difference between the original culture and the target culture. Therefore, in this study, to objectively measure the work engagement level of college students in Korea, the validity and reliability of the Korean version translation/adaptation instrument of the 17-item UWES-S was verified. Therefore, we would like to discuss the results of this study as follows.

When developing the UWES instrument, the model fit for three factors using the multiple group method was χ^2^ = 1859.93, df = 232, GHI = 0.82, AGFI = 0.77, RMSEA = 0.08, NFI = 0.86, NNFI = 0.85, CFI = 0.87 [7]. The model fit for the Spanish version of the 17-item UWES-S was χ^2^ = 505.80, df = 116, TLI = 0.89, CFI = 0.90, RMSEA = 0.07. The model fit of the Portuguese version of the 17-item UWES-S was χ^2^ = 616.41, df = 116, TLI = 0.87, CFI = 0.89, RMSEA = 0.08. The model fit of the Dutch version of the 17-item UWES-S was χ^2^ = 473.03, df = 116, TLI = 0.73, CFI = 0.77, and RMSEA = 0.10 [8]. As a result of referring to the modification index in the results of this study, χ^2^ = 294.858 (df = 114), *p* < 0.001, CFI = 0.925, TLI = 0.910, SRMR = 0.045, and RMSEA = 0.081, thus indicating satisfactory levels. The results confirm that the Korean version of the 17-item UWES-S has the same theoretical structure as previous research and has a similar level of model fit. In particular, the GFI and CFI of this study were 0.90 or higher, and therefore higher than the model fit levels of the Spanish, Portuguese, and Dutch versions of UWES-S, as well as a higher fit than previous studies, thereby indicating that a model close to the optimal was extracted. 

A 9-item UWES for the 17-item UWES has been proposed. As a result of referring to the modification index in the results of this study, χ^2^ = 1545.33 (df = 64), *p* < 0.001, CFI = 0.95, GFI = 0.96, RMSEA = 0.05, thus indicating satisfactory levels. The 9-item UWES has acceptable psychometric properties and the instrument can be used in studies on positive organizational behavior [2]. However, studies on the appropriateness and validity of the Korean version of the 9-item UWES have not yet been conducted. In the future, following this study, it is judged that it will be necessary to proceed with further research.

In addition, in Sri Lanka, the fitness of an instrument consisting of 16 items was performed by removing one item deemed inappropriate in item correlation from the initial 17 items [9]. In the case of India, the last nine items of the UWES-S instrument were selected for the most optimal model [10], and in Japan, the last 14 items of the UWES-S instrument were selected for the most optimal model [11]. However, the current study is meaningful because it consists of an instrument that includes all 17 items of UWES-S, which further suggests the need for a validity and reliability study for the Korean version of the UWES-S.

The r of the Korean version of the 17-item UWES-S of this study was –0.344 (*p* < 0.01), as a result of analyzing the correlation with the Korean academic burnout scale developed and validated by Lee et al. [20]. Thus, convergent validity was supported. The Korean academic burnout scale was developed to measure the academic burnout of Korean college students, and student work engagement plays an important role in burnout. Therefore, in many studies related to academic engagement, the convergent validity was verified through the correlation test with the academic engagement instrument for convergent validity. For example, in correlation with the Utrecht Burnout Scale, the entire instrument and its sub-domains of Vigor, Dedication, and Absorption had a negative correlation with Exhaustion, Cynicism, and Reduced Professional Efficacy, which are sub-items of the Burnout scale, and have a negative correlation coefficient. The values were included within the median value and range [7] for the 17 items in correlation with Exhaustion, Cynicism, and Reduced Efficacy, which are sub-items of the MBI-SS instrument. All *p* of the Spanish version of UWES-S < 0.01 showed a statistically significant negative correlation, and both the Portuguese and Dutch versions showed a negative correlation, but only Reduced Efficacy was statistically significant [8]. Therefore, it can be seen that the convergent validity of the instrument developed in this study was very high. Therefore, the instrument developed in this study can be used to reflect psychological, social, and cultural differences of Korea. This study used an academic burnout scale targeting Korean students as an instrument for developing an instrument for Korean college students.

When developing the UWES instrument, the reliability was 0.82 for Vigor, 0.89 for Dedication, 0.83 for Absorption, and 0.93 for Total [7]. In the case of UWES-S, the Spanish version showed Cronbach’s α in all subregions = 0.79–0.94; this was 0.71–0.87 for the Portuguese version, and 0.83–0.91 for the Dutch version [8]. However, in this study, Cronbach’s α was 0.83–0.94, which was similar to the previous study. Moreover, in this study, McDonald’s omega and composite reliability values were also 0.82–0.941 and 0.833–0.941; thereby, the reliability of the instrument developed in this study was increased. 

As such, the validity and reliability of the Korean version of the UWES-S instrument were confirmed through this study. This will be helpful in understanding the work engagement of domestic nursing college students and comparing the work engagement of college students in other majors or overseas. Furthermore, based on the level of academic engagement, this instrument will contribute to developing a curriculum that is better suited to student characteristics, setting appropriate goals, and designing a self-directed learning process. These results can be used as basic data to determine a strategy for improving work engagement in college students.

Meanwhile, this study faced several limitations in the process of verifying the validity and reliability of the Korean version of the UWES-S. First, although we collected data using a random sampling method, the sample was recruited from one metropolitan city and all the participants of this study were undergraduate nursing students, which could limit the generalizability of the study findings. Second, the model fit of the KUWES-S was acceptable in terms of CFI, TLI, and SRMR, but the fit index of RMSEA was slightly high, with a score of 0.081 in these findings. Further validation studies are needed using a larger sample of college students with various majors. 

## 5. Conclusions

This study attempted to translate the 17-item UWES-S into the Korean language and test its validity and reliability to evaluate the level of academic enthusiasm of domestic college students, and thus provide basic data for the development of effective learning strategies. As a result of the reliability test for construct validity and convergent validity for the Korean version of the 17-item UWES-S instrument, the validity and reliability of the 17 items were high and appropriate. Therefore, the Korean version of the 17-item UWES-S can be used as an instrument to measure learning-related work engagement in Korean nursing students, evaluate the profile of the learning process, and determine the status of students.

## Figures and Tables

**Table 1:** Item descriptive analysis and internal consistency reliability of the KUWES-S (*N* = 248).

Subscales	Mean	SD	Skewness	Kurtosis	Factor Loadings	Item-Total Correlation in Subscale	Item-Total Correlation in Total Scale
Vigor (6 items)							
1. When I’m doing my work as a student, I feel bursting with energy.	2.83	1.10	−0.105	0.66	0.687	0.654	0.697
4. I feel energetic and capable when I’m studying or going to class.	2.67	1.07	0.13	0.07	0.700	0.679	0.707
8. When I get up in the morning, I feel like going to class.	2.09	1.24	0.58	0.16	0.632	0.609	0.629
12. I can continue studying for very long periods at a time.	3.13	1.14	<0.01	0.04	0.676	0.555	0.634
15. I am very resilient, mentally, as far as my studies are concerned	2.82	1.18	0.13	−0.135	0.790	0.718	0.760
17. As far as my studies are concerned, I always persevere, even when things do not go well.	2.82	1.21	0.03	−0.034	0.609	0.557	0.578
Dedication (5 items)							
2. I find my studies full of meaning and purpose.	3.59	1.15	0.09	−0.094	0.672	0.658	0.615
5. I am enthusiastic about my studies.	3.08	1.14	−0.142	0.08	0.820	0.732	0.768
7. I find my studies full of meaning and purpose.	2.61	1.19	0.09	−0.094	0.783	0.716	0.741
10. I am proud of my studies.	3.43	1.25	−0.257	0.03	0.749	0.671	0.681
13. To me, my studies are challenging.	2.97	1.26	−0.261	0.03	0.715	0.637	0.698
Absorption (6 items)							
3. Time flies when I am studying.	3.40	1.23	0.12	0.11	0.576	0.514	0.554
6. When I am studying, I forget everything else around me.	2.65	1.11	0.10	−0.102	0.649	0.587	0.637
9. I feel happy when I am studying intensely.	2.87	1.32	0.02	0.04	0.734	0.645	0.724
11. I am immersed in my studies.	3.17	1.13	−0.134	0.49	0.798	0.684	0.772
14. I get carried away when I am studying.	2.52	1.13	0.13	0.01	0.690	0.667	0.699
16. It is difficult to detach myself from my studies	2.12	1.35	0.50	0.53	0.583	0.531	0.575

**Table 2:** Fit indices from the CFA model of the KUWES-S (*N* = 248).

Model	*χ* ^2^	*df*	*p*	CFI	TLI	SRMR	RMSEA	*p* (RMSEA < 0.05)	RMSEA 90% CI
3-factor model before modification	357.578	116	<0.001	0.901	0.884	0.048	0.092	<0.001	0.08–0.103
3-factor model after modification	294.858	114	<0.001	0.925	0.910	0.045	0.081	<0.001	0.069–0.092

**Table 3:** Convergent validity of relationships in the KUWES-S with academic burnout (*N* = 248).

	Vigor	Dedication	Absorption	Burnout
	r (*p*)			
Academic Engagement (overall)	0.943 (<0.001)	0.941 (<0.001)	0.955 (<0.001)	−0.344 (<0.001)
Vigor	1	0.837 (<0.001)	0.864 (<0.001)	−0.350 (<0.001)
Dedication	0.837 (<0.001)	1	0.853 (<0.001)	−0.337 (<0.001)
Absorption	0.864 (<0.001)	0.853 (<0.001)	1	−0.290 (<0.001)
Academic Burnout	−0.350 (<0.001)	−0.337 (<0.001)	−0.290 (<0.001)	1

**Table 4:** Reliability indices for overall and each subscale.

Reliability Index	Cronbach’s Alpha	McDonald’s Omega	Composite Reliability
Overall	0.940	0.941	0.941
Vigor	0.846	0.839	0.840
Dedication	0.863	0.864	0.865
Absorption	0.830	0.830	0.833

## Data Availability

The Data presented in this study are avaliable within the article, according to MDPI Reasarch Data Policies at https://www.mdpi.com/ethics.

## Appendix A

**Item Number**	**English Version of the 17-Item UWES-S (2002)**	**Korean Version of the 17-Item UWES-S**
1	When I’m doing my work as a student, I feel bursting with energy.	나는 학생으로서 공부할 때, 에너지가 솟구침을 느낀다.
2	I find my studies full of meaning and purpose.	나는 나의 학업의 의미와 목적이 뚜렷하다고 생각한다.
3	Time flies when I am studying.	나는 공부할 때 시간이 빨리 지나간다.
4	I feel energetic and capable when I’m studying or going to class.	나는 공부하거나 수업을 들으러 갈 때 활력과 자신감을 느낀다.
5	I am enthusiastic about my studies.	5. 나는 공부에 대한 열정이 있다.
6	When I am studying, I forget everything else around me.	나는 공부할 때, 내 주위의 다른 일들은 모두 잊어버린다.
7	My studies inspire me.	학업은 나에게 영감을 불어넣어 준다.
8	When I get up in the morning, I feel like going to class.	나는 아침에 일어나면 학교에 가고 싶다.
9	I feel happy when I am studying intensely.	나는 열심히 공부할 때 행복감을 느낀다
10	I am proud of my studies.	나는 내 공부에 대해 자부심을 느낀다.
11	I am immersed in my studies.	나는 공부에 몰두한다.
12	I can continue studying for very long periods at a time.	나는 한번 공부를 시작하면 장기간 계속해서 할 수 있다
13	To me, my studies are challenging.	공부는 나에게 도전감을 준다.
14	I get carried away when I am studying	나는 공부할 때 내 자신을 잊을 정도로 열중한다.
15	I am very resilient, mentally, as far as my studies are concerned.	나는 공부에 관한 한, 정신적으로 잘 이겨낸다.
16	It is difficult to detach myself from my studies.	공부를 안하는 것은 나에게 힘든 일이다.
17	As far as my studies are concerned I always persevere, even when things do not go well.	나는 공부에 관한 한, 일이 잘 안 풀릴 때에도 항상 참고 견딘다.
